# Spiral Bevel Gears Face Roughness Prediction Produced by CNC End Milling Centers

**DOI:** 10.3390/ma11081301

**Published:** 2018-07-27

**Authors:** Álvaro Álvarez, Amaia Calleja, Mikel Arizmendi, Haizea González, Luis Norberto Lopez de Lacalle

**Affiliations:** 1 Ibarmia, Polígono Industrial Etxesaga, s/n, 20720 Azkoitia, Gipuzkoa, Spain; alvaro.alvarez@ibarmia.com; 2 Department of Mechanical Engineering, University of the Basque Country (UPV/EHU), Nieves Cano 12, 01006 Vitoria, Spain; 3 Department of Mechanical Engineering, TECNUN-Universidad de Navarra, Paseo de Manuel Lardizabal 13, 20018 Donostia-San Sebastián, Spain: marizmendi@tecnun.es; 4 Department of Mechanical Engineering, University of the Basque Country (UPV/EHU), Plaza Ingeniero Torres Quevedo 1, 48013 Bilbao, Spain; haizea.gonzalez@ehu.eus; 5 CFAA—University of the Basque Country (UPV/EHU), Parque Tecnológico de Zamudio 202, 48170 Bilbao, Spain; norberto.lzlacalle@ehu.eus

**Keywords:** gear manufacturing, roughness model, multitasking machines/multiprocess machines

## Abstract

The emergence of multitasking machines in the machine tool sector presents new opportunities for the machining of large size gears and short production series in these machines. However, the possibility of using standard tools in conventional machines for gears machining represents a technological challenge from the point of view of workpiece quality. Machining conditions in order to achieve both dimensional and surface quality requirements need to be determined. With these considerations in mind, computer numerical control (CNC) methods to provide useful tools for gear processing are studied. Thus, a model for the prediction of surface roughness obtained on the teeth surface of a machined spiral bevel gear in a multiprocess machine is presented. Machining strategies and optimal machining parameters were studied, and the roughness model is validated for 3 + 2 axes and 5 continuous axes machining strategies.

## 1. Introduction

Large size spiral bevel gears are frequently used in applications [1,2] that require smooth and silent high-power transmission. This is the case for equipment dedicated to thermal energy generation, ship propulsion systems, wind turbines or power transmission in the aeronautical sector, among many others. Nowadays, there is a continuous demand for energy, and consequently, there has been an increase in the amount of equipment dedicated to energy generation and its components, such as large sized spiral gear. Traditionally, these types of gears have been manufactured with specific gear cutting machines. There are different methods for traditional gear cutting, for example, some of the most commonly used are: (1) gear hobbing with perimeter cut (Gleason) [3]; (2) continuous generation by spiral hobbing with perimeter cut (Cyclo-Palloid from Klingelnberg and Oerlikon) [4]; and (3) continuous generation by spiral hobbing with conic type cut (Palloid from Klingelnberg) [5].

However, the eruption in the market of multitasking or multiprocess machines [6], and the continuous improvement experienced in the area of numerical controls and CAM software, has led to the appearance of a suitable medium for the manufacturing of these complex geometric elements in general purpose machines and with standard tools [7,8]. This type of technology is especially interesting for the manufacture of high module gears (4–12 mm), where it is not so common to find specific gear cutting machines as in the case of lower value modules. The use of standard tools is also an advantage given the reduction in both cost and delivery times, which are parameters of vital importance in production. Special tools for gear manufacturing are also available [9], thus providing flexible alternatives for producing small or medium batches of large bevel gears using a five-axis machine and a disk tool cutting method. This methodology also allows the manufacture of gears of varied geometries, for example, straight gears, helical gears, double helical gear, bevel gears and hypoid gears. The manufacture of gears in multitasking machines [10,11,12,13,14,15,16,17] is seen as an increasingly widespread solution, especially given their high flexibility [18]. Four-axis [19] and 5-axis [20,21] CNC machining can be performed for spiral bevel gears manufacturing. Some of the advantages of this method include an increase in the versatility of the manufacturing process, both in terms of typology and size, allowing the realization of arbitrary modifications of the different gear teeth. Surface quality and the structure of the materials are also important for gear life, as studied in [21]. In order to guarantee the quality of the manufactured components and gear contact [22,23,24,25,26], the machining process of the gear surfaces requires special attention. Surface morphology [27] will determine machining strategies, making it possible to machine gear sculptured surfaces [9], classified as developable ruled surfaces [28], with flank milling strategies [29,30].

On the other hand, gears, and more specifically spiral bevel gears (bevel gears with helical teeth), are geometrically complex components. Once the feasibility of manufacturing these components in multi-axis general-purpose machines has been demonstrated, it is necessary to evaluate whether the number of the machines axes involved in the machining strategy influence the resulting gear surface quality. Since the transmission of movement and power between different axes is the main function of this type of gears, there is a greater contact surface between the pinion and gear compared to a pair of straight bevel gears. Due to the helix angle, spiral gears work in a gradual way, operating with greater smoothness and more silently, allowing work at higher speed ranges. However, this type of gear presents a greater sensitivity to contact errors than other types of gears.

Therefore, in order to ensure good gear contact, the surface roughness parameter is a parameter to be considered and studied in the manufacture of spiral bevel gears by multi-process machines. Optimal surface roughness values ensure good contact [31], which is translated into the correct transmission of both movement and power, increasing the useful life of the element. It is worth mentioning that both excessive surface roughness and polished surfaces are harmful for gear contact. On one hand, gear rough surfaces influence component life, and, on the other hand, gear polished surfaces hinder proper lubrication.

In this work, a predictive model of surface roughness for spiral bevel gears manufactured by multiprocess machines with ball end mills was developed and validated. The model estimates surface topography for each gear surface based on parameters such as tool inclination and orientation, the geometrical cutting parameters, and mill feed and speed values. The gear machining finishing process is optimized by the simulation of different machining conditions. Thus, it is not necessary to perform trial and error tests, which results in cost and time savings. This optimized process also adjusts cutting parameters depending on the required surface quality, without having to machine a greater number of passes than strictly necessary. This also reduces machining time and tool life.

## 2. Spiral Bevel Gears Manufacturing Process in Multitasking Machines

The gear manufacturing process consists of several stages. First, the geometry of the component, which directly influences the subsequent manufacturing stages, is defined.

### 2.1. Design

There are different options for the design of gear geometry [32,33,34]: Standard CAD/CAM software (CatiaV5, Siemens NX12), specific gear design module inside standard CAD/CAM software (GearTrax module for Solid Edge, SolidWorks and Inventor), specific CAD/CAM software (EUKLID), software developed by machine tool manufacturers (gear MILL from DMG-MORI, GearPro from Mag), and, software developed by tool manufacturers (InvoMilling and Up-Gear Technology from Sandvik).

In this particular case, the “3d spiral bevel gear software” was used for the design of the gear geometry. The main reason for choosing this software was the reduced cost of the license in comparison to other software. Specifically, this program allows the design of the spiral bevel geometry, with the option to choose between Gleason and Klingelnberg manufacturing methods.

A spiral bevel gear geometry was selected (Table 1) according to the Gleason method, since it is the most used method. The objective was to choose a complex and large dimension geometry to validate the capacity of general purpose multiprocessing machines for gear manufacturing.

The selected material is a commonly used steel for manufacturing gears, F-1550 (18CrMo4) (C 0.186%, Si 0.259%, Mn 0.805%, P 0.011%, S 0.028%, Cr 1.071%, Mo 0.155%), and it reaches values up to 47 HRC (Rockwell Scale of Hardness, part C).

First, geometric parameters were introduced into the design software (module, gear ratio, gear direction, teeth number, pressure angle, etc.). With this information, the software generated the geometry of one of the teeth, from which the three-dimensional gear was generated.

### 2.2. Manufacturing

Once the geometry was obtained and analyzed, the machining strategies were designed. In this case, the CAM software used was NX10 from Siemens.

### 2.3. Equipment—Multiprocess Machine

**Figure 1:**
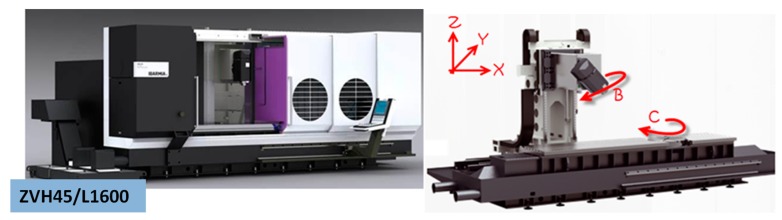
Ibarmia ZVH45/L1600 ADD + PROCESS kinematic.

A multi-process machine ZVH38/L1600 (Figure 1) from Ibarmia (Azkoitia, Spain) was used for gear manufacturing. The machine includes turning and milling capabilities by the integration of 3 linear axes (X, Y, Z) in a mobile column and 2 rotary axes, one of them in a rotating head (B) and the other in a rotary table (C). The main advantage of multitasking machines is that the number of machines and setups required for workpiece manufacturing are reduced and often limited to one.

### 2.4. Machining Strategies

**Figure 2:**
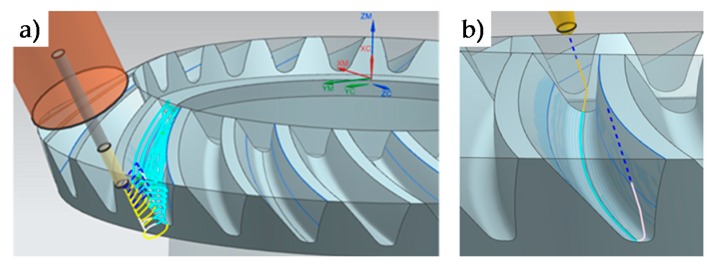
R-I (**a**) and R-II (**b**) roughing strategies.

Machining strategies were programmed with Siemens^®^ NX manufacturing module. First of all, the roughing operation was programmed with the aim of reducing machining time, chip thickness and cutting forces [18] as much as possible and obtaining a near to net shape geometry. As can be seen in Table 2, the roughing operation (Figure 2) consisted of two different steps. First, a cavity mill strategy was performed, and then, a variable contour strategy was used in order to obtain a near to net shape geometry for finishing strategies. In R-I a frontal mill was used and in R-II a conical mill.

**Figure 3:**
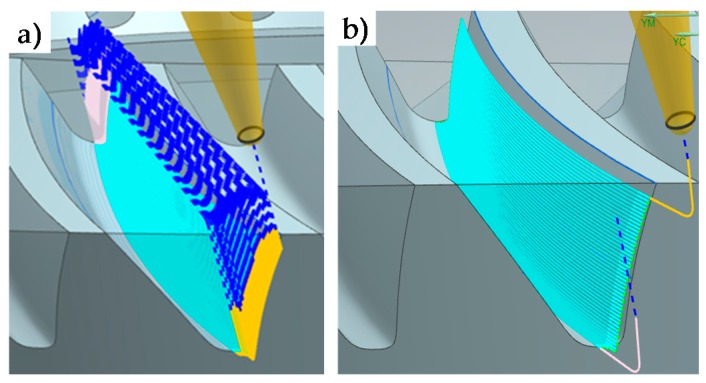
Finishing strategies. F-I (**a**) 3 + 2 finishing strategy. F-II (**b**) 5 continuous axes finishing strategy.

For finishing operations (Table 3), different strategies and conditions were tested for the gear teeth. Different radial depths and cut patterns (zig and zig-zag) were evaluated, for both 5 continuous axes (F-II) machining operations and 3 + 2 axes (F-I) machining. In the latter machining operation (3 + 2), the tool axis is fixed and perpendicular to the head of each tooth, as can be seen in Figure 3. Machining conditions were S = 11,900 rpm and F = 400 mm/min.

### 2.5. Machining

**Figure 4:**
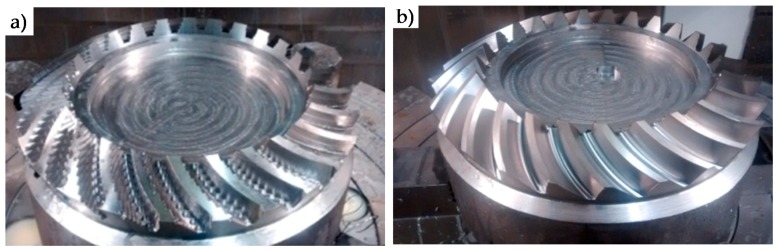
Roughing strategies result (**a**); Finishing strategies result (**b**).

Finally, the gear was machined. After the roughing and finishing operations, the resultant gear is shown in Figure 4. Machined strategies were R-I (cavity mill) for teeth 1–25 and R-II (variable contour) after R-I and for teeth 1–25. For the finishing strategies: F-I for teeth 1–12, and F-II for teeth 13–25.

## 3. Predictive Model of Topography on Gear Flank

The developed model estimates the gear teeth surface topography depending on machining parameters such as tool inclination and orientation, cutting geometric parameters and tool feed and speed values. The model was tested for two different finishing operations; a 5 continuous axes machining operation and a 3 + 2 axes machining operation in order to determine the influence of the machining number of axes on the surface finish.

**Figure 5:**
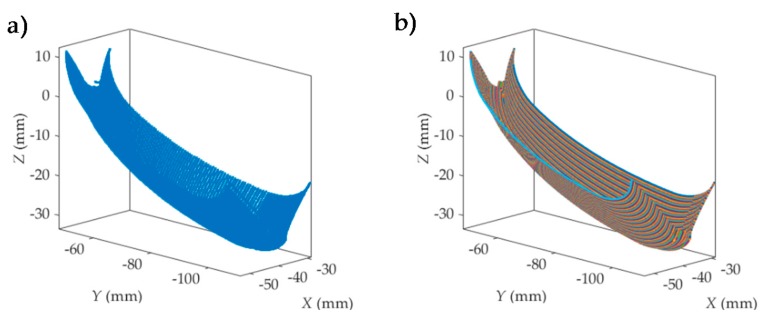
Interdental gap points (**a**) and trajectories (**b**) representation.

**Figure 6:**
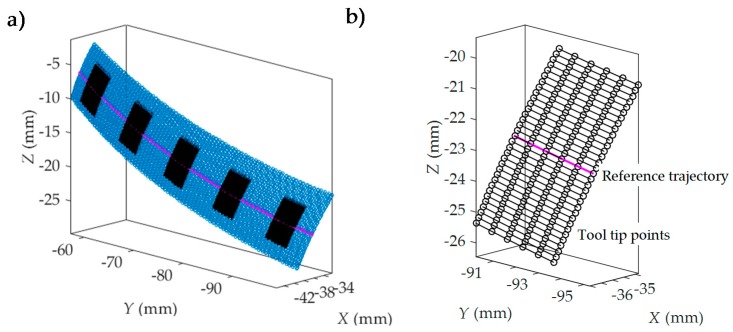
Simulation area (**a**) and reference trajectory points (**b**) for the study definition.

**Figure 7:**
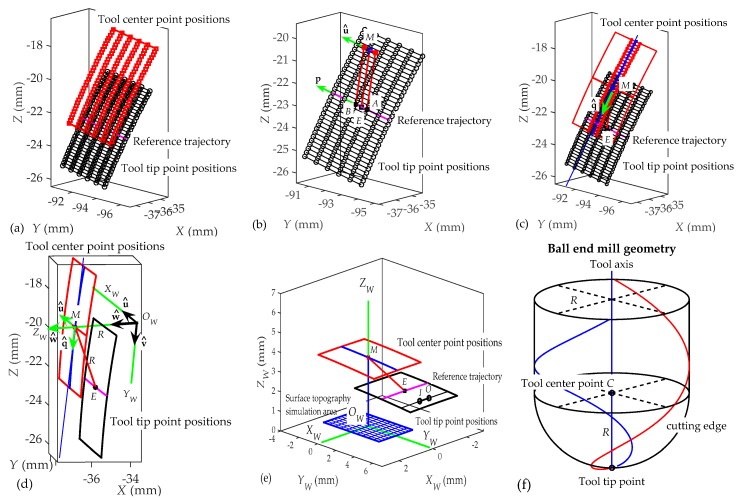
Tool center points (red) in relation to tool tip points (black) and to tool axis orientation and local reference system *O_W_X_W_Y_W_Z_W_* attached to workpiece (gear tooth). (**a**) Tool center points position; (**b**) M point definition. (**c**) Reference trajectory; (**d**) Tool tip and center point positions; (**e**) Reference for the prediction of the topography generated in the surface of gear teeth; (**f**) Ball end mill geometry.

The model follows the following steps:

1. **Interdental gap points and trajectories representation (Figure 5).** In this first step, for each interpolation point the model obtains from the machining program (CL data): the tool tip point position (*x_j_*, *y_j_*, *z_j_*) and the tool axis orientation defined by a direction vector (*u_j_*, *v_j_*, *w_j_*) in the workpiece reference system *XYZ*.
  Milling trajectories can be obtained by means of the coordinates of successive tool tip points. Figure 5a shows the tool tip point positions obtained from the machining program of an interdental gap. In Figure 5b, the milling trajectories are represented. In the next steps, milling trajectories are evaluated after the elimination of initial and final noncutting movements, as shown in Figure 6a.
2. **Simulation area definition (Figure 6).** Secondly, as can be seen in Figure 6a, five simulation areas are defined (in black) in the feed direction. Each of these simulation areas is defined by 6 points in the feed direction and about 30 points in the direction perpendicular to the feed direction. The dimension of the simulation area depends on the programmer’s criteria. The reference trajectory for the study is defined in magenta, corresponding to the one located in the middle of the simulation area. As an example, Figure 6b shows the milling trajectories followed by the tool tip in one of the simulation areas considered in Figure 6a. Black points in each milling trajectory represent the interpolation points given by the machining program for the selected simulation area. Next, the surface topography generated in each simulation area is predicted. In order to achieve this, a local reference system *O_W_X_W_Y_W_Z_W_* is defined for each simulation area (Step 4). First, the positions of the tool center point (*C*) in the selected simulation area are deduced in Step 3.
3. **Tool center position determination (Figure 7).** In order to obtain the coordinates of the tool center point (*C*) (Figure 7f), tool tip point coordinates (*x_j_*, *y_j_*, *z_j_*) and tool axis direction vector (*u_j_*, *v_j_*, *w_j_*) given in the machining program and the ball end mill, radius *R* needs to be taken into account. First, the line passing through the point (*x_j_*, *y_j_*, *z_j_*) and parallel to the vector (*u_j_*, *v_j_*, *w_j_*) is considered by means of the following equation:
  (1) $$\frac{{x-x}_{j}}{u_{j}}=\frac{{y-y}_{j}}{v_{j}}=\frac{{z-z}_{j}}{w_{j}}$$

Next, taking into account that the tool center point (*C*) is located at a distance equal to the tool radius *R* from the tool tip point of coordinates (*x_j_*, *y_j_*, *z_j_*), the coordinates (*x*, *y*, *z*) of the tool center point must fulfill this equation:

(2) $$\sqrt{{\left({x-x}_{j}\right)}^{2}+{\left({y-y}_{j}\right)}^{2}+{\left({z-z}_{j}\right)}^{2}}=R$$

Therefore, in order to obtain the coordinates (*x*, *y*, *z*) of the tool center point when the tool tip point is located at a point of coordinates (*x_j_*, *y_j_*, *z_j_*) and tool axis vector is (*u_j_*, *v_j_*, *w_j_*), Equations (1) and (2) must be solved for *x*, *y* and *z*.

The red points shown in Figure 7a represent the positions of the tool center point (*C*) for the simulation area shown in Figure 6b. The surface generated by the positions of the tool center point approximates to a surface parallel to the tool tip point positions.

4. **Definition of workpiece local coordinate system.** In order to predict the surface topography generated in each simulation area, a local coordinate system *O_W_X_W_Y_W_Z_W_* attached to the workpiece (gear tooth surface) is defined. The definition of this local coordinate system for each simulation area is based on the tool tip point positions and the tool axis orientations given in the machining program for the reference milling trajectory shown in magenta in Figure 6 and Figure 7. The origin *O_W_* and the axis *Z_W_* are the elements of this system that are obtained first. In order to achieve this, a set of auxiliary elements (a point *M* and two unit vectors $\hat{u}$ and $\hat{q}$) is considered.
  Firstly, two interpolation points, named *A* and *B,* which are located in the center of the reference trajectory (Figure 7b) are selected. The middle point *D* of the linear interpolation between points *A* and *B* is considered. The coordinates (*x_D_*, *y_D_*, *z_D_*) of point *D* are calculated as a function of the coordinates (*x_A_*, *y_A_*, *z_A_*) and (*x_B_*, *y_B_*, *z_B_*) of points *A* and *B*:
  (3) $$x_{D}\;=\;\frac{x_{A}+x_{B}}{2}{;\;y}_{D}\;=\;\frac{y_{A}+y_{B}}{2}{;\;z}_{D}\;=\;\frac{z_{A}{+z}_{B}}{2}$$

Taking into account the tool axis orientation when the tool tip goes from point *A* to point *B*, the position of the tool center point when the tool tip point is located at point *D* can be calculated through Equations (1) and (2). Therefore, this position defined in this paper as point *M* (Figure 7b), with coordinates (*x_M_*, *y_M_*, *z_M_*), can be obtained from the resolution of the following equations:

(4) $$\frac{x_{M}{-x}_{D}}{u_{AB}}\;=\;\frac{y_{M}{-y}_{D}}{v_{AB}}\;=\;\frac{z_{M}{-z}_{D}}{w_{AB}}$$

(5) $$\sqrt{{\left(x_{M}{-x}_{D}\right)}^{2}\;+\;{\left(y_{M}{-y}_{D}\right)}^{2}\;+\;{\left(z_{M}{-z}_{D}\right)}^{2}}\;=\;R$$

where (*u_AB_*, *v_AB_*, *w_AB_*) is the direction vector of tool axis, given by the machining program, when the tool tip goes from point *A* to point *B*. The point *M* is calculated for each simulation area and employed for the definition of the local system attached to each simulation area. In this paper, it is assumed that axis *Z_W_* of the local system passes through this point *M*.

Next, the vector defining the direction of axis *Z_W_* is calculated by considering two unit vectors. A unit vector $\hat{u}$ = (*u_x_*, *u_y_*, *u_z_*) parallel to the tool linear motion direction between points *A* and *B* is defined as:

(6) $$\hat{u}=\frac{p}{\left|p\right|}\;\mathrm{where}\;p\;=\;\left(x_{B}{-x}_{A}{,\;y}_{B}{-y}_{A}{,\;z}_{B}{-z}_{A}\right)$$

A second unit vector $\hat{q}$ = (*q_x_*, *q_y_*, *q_z_*) that takes into account the direction at point *M* of the middle points of the milling trajectories selected in the simulation area is also defined (Figure 7c). Once the unit vectors $\hat{u}$ and $\hat{q}$ are obtained, a unit vector $\hat{w}$ = (*w_x_*, *w_y_*, *w_z_*) perpendicular to $\hat{u}$ and $\hat{q}$ is defined as follows:

(7) $$\hat{u}\;=\hat{u}\times \hat{q}$$

This vector $\hat{w}$ is assumed to coincide with the direction of axis *Z_W_* (Figure 7d). In addition, in order to define the position of the origin *O_W_*, it is assumed that this point is located at a distance *R* from the point *M*. Therefore, taking into account that point *O_W_* is also located on a line passing through point *M* and parallel to vector $\hat{w}$, the coordinates (*x_Ow_*, *y_Ow_*, *z_Ow_*) of point *O_W_* are obtained by solving these equations:

(8) $$\frac{x_{\mathrm{Ow}}{-x}_{M}}{w_{x}}\;=\;\frac{y_{\mathrm{Ow}}{-y}_{M}}{w_{y}}\;=\;\frac{z_{\mathrm{Ow}}{-z}_{M}}{w_{z}}$$

(9) $$\sqrt{{\left(x_{\mathrm{Ow}}{-x}_{M}\right)}^{2}\;+\;{\left(y_{\mathrm{Ow}}{-y}_{M}\right)}^{2}+\;{\left(z_{\mathrm{Ow}}{-z}_{M}\right)}^{2}}=R$$

Once the origin *O_W_* and the axis *Z_W_* have been obtained, axes *X_W_* and *Y_W_* are defined. The axis *X_W_* is assumed to have the same direction as the unit vector $\hat{u}$ (Figure 7d). Therefore, the axis *X_W_* coincides with the feed direction of the tool between points *A* and *B*. Finally, the axis *Y_W_* is perpendicular to axes *X_W_* and *Z_W_*. The direction of axis *Y_W_* is defined by a unit vector $\hat{v}$ = (*v_x_*, *v_y_*, *v_z_*) calculated as the cross product between vectors $\hat{w}$ and $\hat{u}$:

(10) $$\hat{v}\;=\;\hat{w}\times \hat{u}$$

Figure 7e shows an equivalent representation of Figure 7d. In Figure 7e, the local system *O_W_X_W_Y_W_Z_W_* is taken as a reference for the prediction of the topography generated in the surface of gear teeth. The surface topography will be simulated in a rectangular area defined along axes *X_W_* and *Y_W_*, as shown in Figure 7e. In order to model the surface roughness, the equations expressing the trajectories of tool cutting edges in this local system are deduced in Step 6 as a function of the ball end mill geometry (Step 5), the tool axis orientation and the milling trajectories followed by the tool in the simulation area. Firstly, the coordinates of points defining the milling trajectories and the direction vector of tool axis orientations expressed in the workpiece system *XYZ* (given by the machining program and obtained in Step 1) must be transformed into the local system *O_W_X_W_Y_W_Z_W_*.

In order to express the coordinates of a point in the local system *O_W_X_W_Y_W_Z_W_* from its coordinates in the workpiece system *XYZ*, a homogeneous transformation matrix **T** is defined as a function of the unit vectors $\hat{u}$, $\hat{v}$ and $\hat{w}$ and the coordinates of the origin *O_W_* in the system *XYZ*. The coordinates (*x_j_*, *y_j_*, *z_j_*) of the tool tip point positions given in the machining program can be expressed in the local system *O_W_X_W_Y_W_Z_W_*, (*x_j_^W^*, *y_j_^W^*, *z_j_^W^*), as:

(11) $$\left[\begin{array}{c} x_{j}\\ y_{j}\\ z_{j}\\ 1 \end{array}\right]\;=\;\left[\begin{array}{cccc} u_{x} & v_{x} & w_{x} & x_{\mathrm{Ow}}\\ u_{y} & v_{y} & w_{y} & y_{\mathrm{Ow}}\\ u_{z} & v_{z} & w_{z} & z_{\mathrm{Ow}}\\ 0 & 0 & 0 & 1 \end{array}\right]\left[\begin{array}{c} x_{j}^{W}\\ y_{j}^{W}\\ z_{j}^{W}\\ 1 \end{array}\right]\;=\;T\left[\begin{array}{c} x_{j}^{W}\\ y_{j}^{W}\\ z_{j}^{W}\\ 1 \end{array}\right]\;\to \;\left[\begin{array}{c} x_{j}^{W}\\ y_{j}^{W}\\ z_{j}^{W}\\ 1 \end{array}\right]\;={\;T}^{-1}\left[\begin{array}{c} x_{j}\\ y_{j}\\ z_{j}\\ 1 \end{array}\right]$$

Similarly, a direction vector (*u_j_*, *v_j_*, *w_j_*) given in the machining program can be expressed in the local system *O_W_X_W_Y_W_Z_W_*, (*u_j_^W^*, *v_j_^W^*, *w_j_^W^*) by means of a matrix **T**_1_ depending on the unit vectors $\hat{u}$, $\hat{v}$ and $\hat{w}$

(12) $$\left[\begin{array}{c} u_{j}\\ v_{j}\\ w_{j}\\ 1 \end{array}\right]\;=\;\left[\begin{array}{cccc} u_{x} & v_{x} & w_{x} & 0\\ u_{y} & v_{y} & w_{y} & 0\\ u_{z} & v_{z} & w_{z} & 0\\ 0 & 0 & 0 & 1 \end{array}\right]\left[\begin{array}{c} u_{j}^{W}\\ v_{j}^{W}\\ w_{j}^{W}\\ 1 \end{array}\right]{\;=\;T}_{1}\left[\begin{array}{c} u_{j}^{W}\\ v_{j}^{W}\\ w_{j}^{W}\\ 1 \end{array}\right]\;\to \left[\begin{array}{c} u_{j}^{W}\\ v_{j}^{W}\\ w_{j}^{W}\\ 1 \end{array}\right]{\;=\;T}_{1}{}^{-1}\left[\begin{array}{c} u_{j}\\ v_{j}\\ w_{j}\\ 1 \end{array}\right]$$

Once the tool tip point positions and the tool axis orientations are expressed in the local system *O_W_X_W_Y_W_Z_W_*, the equations for the cutting edge trajectory are deduced. In order to achieve this, the geometry of the tool cutting edges is first modeled.

**Figure 8:**
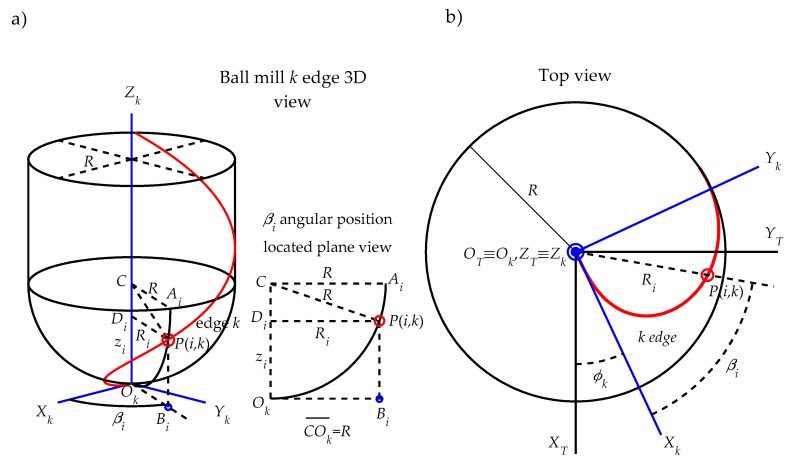
Ball end mill geometry (**a**) and *k* edge geometry definition (**b**).

5. **Tool geometric modelization (Figure 8).** The roughness model is developed for a ball end mill geometry, whose behavior resembles that of a conical tool that only cuts with the spherical area of the tool tip for gear teeth finishing trajectories. Figure 8a shows schematically the 3D geometry of a ball end mill of radius *R* and helix angle *i*_0_. For simplicity, only one of the edges is represented in the figure (Figure 8b), but the developed model is generalized for a *N_t_* edge mill. It is assumed that the cutting edge represented in this figure represents one of the edges of the milling cutter, which is referred to as a *k* edge, where *k* = 1, 2, ..., *N_t_*. To define the position of a point located on the edge *k*, a reference system *O_T_X_T_Y_T_Z_T_* attached to the ball end mill is defined:
  ■ The reference system origin *O_T_* is located on the tool tip being coincident with the tool axis
  ■ The axis *Z_T_* corresponds to the tool axis
  ■ The axis *X_T_* is radial and tangent to edge 1 projection in the plane containing point *O_T_* and perpendicular to *Z_T_* axis
  ■ The axis *Y_T_* is perpendicular to axes *X_T_* and *Y_T_* forming a right-handed system.

The position angle $\varphi_{k}$ of cutting edge *k* with respect to axis *X_T_* can be expressed as:

(13) $$\varphi_{k}\;=\;\frac{2\pi }{N_{t}}(k-1)$$

The position of a cutting edge point *P*(*i*, *k*), located at a height $z_{i}$ on the edge *k*, can be written in the tool reference system *O_T_X_T_Y_T_Z_T_* as a function of (a) the radius $R_{i}$ and the position angle $\beta_{i}$ of cutting edge point at height $z_{i}$ and (b) the position angle $\varphi_{k}$ of edge *k* as follows:

(14) $$x_{P\left(i,k\right)}^{T}{\;=\;R}_{i}{\cdot \cos(\beta }_{i}+\varphi_{k})$$

(15) $$y_{P\left(i,k\right)}^{T}{\;=\;R}_{i}{\cdot \sin(\beta }_{i}+\varphi_{k})$$

(16) $$z_{P\left(i,k\right)}^{T}{\;=\;z}_{i}$$

where, from ball end mill geometry, the radius $R_{i}$ and the position angle $\beta_{i}$ are:

(17) $$R_{i}\;=\;\sqrt{{2Rz}_{i}-{\left(z_{i}\right)}^{2}}$$

(18) $$\beta_{i}\;=\;z_{i}\cdot \tan\left(i_{0}\right)/R\;$$

Next, the equations of trajectories followed by cutting edge points are deduced.

**Figure 9:**
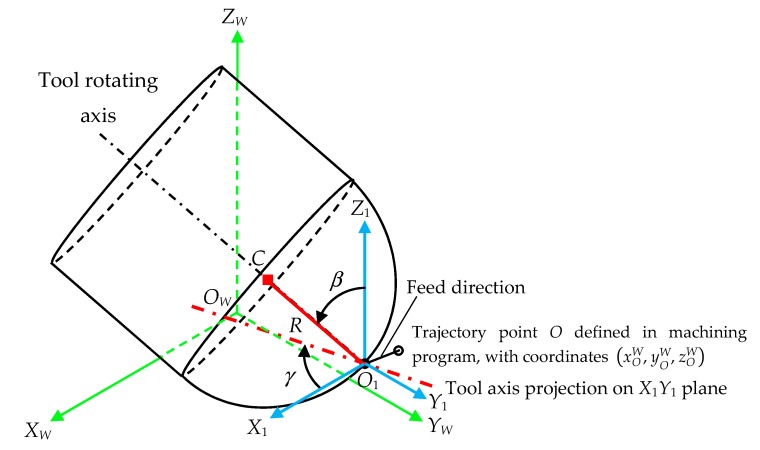
5-axis milling scheme with tool tilt angle *β* and lead angle *γ*.

**Figure 10:**
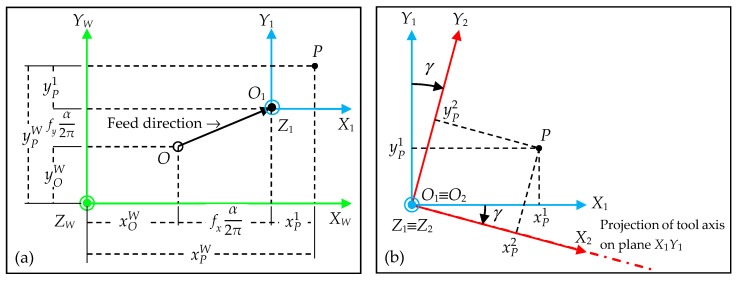

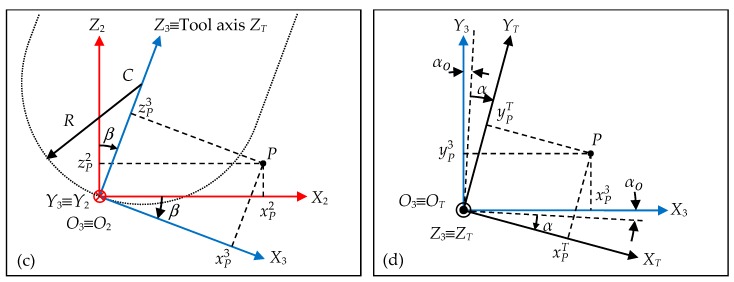
Definition of auxiliary systems *O*_1_*X*_1_*Y*_1_*Z*_1_ (**a**,**b**); *O*_2_*X*_2_*Y*_2_*Z*_2_ (**c**) and *O*_3_*X*_3_*Y*_3_*Z*_3_ (**d**).

6. **Tool axis orientation and points trajectories determination (Figure 9 and Figure 10).** In this step, the trajectory followed by any cutting edge point in a five-axis milling operation is expressed as a function of cutting parameters, tool axis orientation and milling trajectories defined in the machining program. The cutting parameters are the feed value (F) in mm/min and the spindle speed (S) in rpm. Therefore, the tool feed in mm per revolution can be calculated as:
  (19) $$f\;\left(\frac{\mathrm{mm}}{\mathrm{rev}}\right)\;=\;\frac{F\left(\mathrm{mm}/\min\right)}{S\left(\mathrm{rpm}\right)}$$

In order to define the cutting trajectories, as an example, the linear motion shown in Figure 7e, when the tool tip point goes from a point *O* to a point *I* in a milling trajectory of the simulation area, is considered. The procedure presented below is carried out in every section of the milling trajectories located inside the simulation area.

From the machining program, the coordinates (*x_O_*, *y_O_*, *z_O_*) and (*x_I_*, *y_I_*, *z_I_*) of points *O* and *I* and the direction vector (*u_OI_*, *v_OI_*, *w_OI_*) of tool axis orientation during this linear motion can be known. By means of the matrices given by Equations (11) and (12), the coordinates (*x_O_^W^*, *y_O_^W^*, *z_O_^W^*) and (*x_I_^W^*, *y_I_^W^*, *z_I_^W^*) of these points and the direction vector (*u_OI_^W^*, *v_OI_^W^*, *w_OI_^W^*) can be expressed in the local system *O_W_X_W_Y_W_Z_W_* attached to each simulation area.

Taking into account the feed direction of the ball end mill along the linear motion between points *O* and *I*, feed values *f_x_*, *f_y_* and *fz* along axes *X_W_*, *Y_W_* and *Z_W_* can be defined. In order to achieve this, a unit vector $\hat{r}$ = (*r_x_*, *r_y_*, *r_z_*) that considers the tool feed direction between points *O* and *I*, is defined as a function of their coordinates:

(20) $$\hat{r}=\frac{q}{\left|q\right|}\;\mathrm{where}\;q\;=\;\left(x_{I}^{W}{-x}_{O}^{W}{,\;y}_{I}^{W}{-y}_{O}^{W}{,\;z}_{I}^{W}{-z}_{O}^{W}\right)$$

The components *f_x_*, *f_y_* and *fz* of the tool feed can be expressed as:

(21) $$f_{x}{\;=\;fr}_{x}$$

(22) $$f_{y}{\;=\;fr}_{y}$$

(23) $$f_{z}{\;\;=\;fr}_{z}$$

By means of feed values *f_x_*, *f_y_* and *fz*, the position of the tool tip point along the linear displacement between points *O* and *I* is expressed as a function of the tool rotation angle *α* in radians. In Figure 9, it is assumed that the tool tip point is located at a point *O*_1_ whose coordinates are:

(24) $$x_{O1}^{W}{\;=\;x}_{O}^{W}{\;+\;f}_{x}\frac{\alpha }{2\pi }$$

(25) $$y_{O1}^{W}{\;=\;y}_{O}^{W}{\;+\;f}_{y}\frac{\alpha }{2\pi }$$

(26) $$z_{O1}^{W}{\;=\;z}_{O}^{W}{\;+\;f}_{z}\frac{\alpha }{2\pi }$$

where the tool rotation angle *α* goes from 0 to *α_I_-α_O_*, being *α_I_* and *α_O_*, the tool rotation angles when the tool tip point is located at points *I* and *O*, respectively.

In order to simplify the deduction of cutting edge trajectories, the tool axis orientation is defined in this step by means of two angles, which are represented in Figure 9: a tilt angle *β* and a lead angle *γ*.

■ The tilt angle *β* is defined as the angle between the tool rotating axis and the axis *Z*_1_, which is parallel to axis *Z_W_*.
■ The lead angle *γ* is the angle between the projection of the tool rotating axis into plane *X*_1_*Y*_1_ (which is parallel to plane *X_W_Y_W_*) with respect to axis *X_W_* direction.

These angles *β* and *γ* can be written as function of the tool axis direction vector (*u_OI_^W^*, *v_OI_^W^*, *w_OI_^W^*):

(27) $${\beta \;=\;\mathrm{acos}(w}_{OI}^{W})$$

(28) $$\begin{array}{l} \gamma =a\tan(-\frac{v_{OI}^{W}}{u_{OI}^{W}}) \end{array}$$

In order to obtain the equations expressing the trajectory of cutting edge points in the local system *O_W_X_W_Y_W_Z_W_* from their coordinates in the tool system *O_T_X_T_Y_T_Z_T_*, three auxiliary systems *O*_1_*X*_1_*Y*_1_*Z*_1_, *O*_2_*X*_2_*Y*_2_*Z*_2_ and *O*_3_*X*_3_*Y*_3_*Z*_3_, shown in Figure 10, are defined as a function of:

- The current position of the tool tip point given by the coordinates of point *O*_1_ (Figure 9). A translation of system *O*_1_*X*_1_*Y*_1_*Z*_1_ with respect to system *O_W_X_W_Y_W_Z_W_* (Figure 10a) is considered. The system *O*_1_*X*_1_*Y*_1_*Z*_1_ is shifted in *X_W_*, *Y_W_* and *Z_W_* by distances *x_O_*_1_*^W^*, *y_O_*_1_*^W^* and *z_O_*_1_*^W^* respectively. The homogeneous transformation matrix **T***_W_*_1_(*f_x_*, *f_y_*, *f_z_*, *α*) of this translation in *X_W_*, *Y_W_* and *Z_W_* is:
  (29) $$T_{W1}\left(f_{x},\;f_{y},\;f_{z},\;\alpha \right)=\left[\begin{array}{cccc} 1 & 0 & 0 & x_{O1}^{W}\\ 0 & 1 & 0 & y_{O1}^{W}\\ 0 & 0 & 1 & z_{O1}^{W}\\ 0 & 0 & 0 & 1 \end{array}\right]=\left[\begin{array}{cccc} 1 & 0 & 0 & x_{O}^{W}{+f}_{x}\alpha /\left(2\pi \right)\\ 0 & 1 & 0 & y_{O}^{W}{+f}_{y}\alpha /\left(2\pi \right)\\ 0 & 0 & 1 & z_{O}^{W}{+f}_{z}\alpha /\left(2\pi \right)\\ 0 & 0 & 0 & 1 \end{array}\right]$$
- The lead angle *γ* (Figure 10b). The system *O*_2_*X*_2_*Y*_2_*Z*_2_ is rotated by an angle *γ* about axis *Z*_1_ in clockwise direction. The homogeneous transformation matrix **T**_12_(*γ*) of this rotation about *Z*_1_-axis is:
  (30) $$T_{12}\left(\gamma \right)=\left[\begin{array}{cccc} \cos\left(\gamma \right) & \sin\left(\gamma \right) & 0 & 0\\ -\sin\left(\gamma \right) & \cos\left(\gamma \right) & 0 & 0\\ 0 & 0 & 1 & 0\\ 0 & 0 & 0 & 1 \end{array}\right]$$
- The tilt angle *β* (Figure 10c). The system *O*_3_*X*_3_*Y*_3_*Z*_3_ is rotated by an angle β about axis *Y*_2_ in a clockwise direction. The homogeneous transformation matrix **T**_23_(*β*) of this rotation about *Y*_2_-axis is:
  (31) $$T_{23}\left(\beta \right)=\left[\begin{array}{cccc} \cos\left(\beta \right) & 0 & \sin\left(\beta \right) & 0\\ 0 & 1 & 0 & 0\\ -\sin\left(\beta \right) & 0 & \cos\left(\beta \right) & 0\\ 0 & 0 & 0 & 1 \end{array}\right]$$
- The rotation angle *α* (Figure 10d). The tool system *O_T_X_T_Y_T_Z_T_* is rotated by an angle *α* about axis *Z*_3_ in clockwise direction. The homogeneous transformation matrix **T**_3*T*_(*α*) of this rotation about *Z*_3_-axis is:
  (32) $$T_{3T}\left(\alpha \right)=\left[\begin{array}{cccc} \cos\left({\alpha +\alpha }_{O}\right) & \sin\left({\alpha +\alpha }_{O}\right) & 0 & 0\\ -\sin\left({\alpha +\alpha }_{O}\right) & \cos\left({\alpha +\alpha }_{O}\right) & 0 & 0\\ 0 & 0 & 1 & 0\\ 0 & 0 & 0 & 1 \end{array}\right]$$
  where *α_O_* is the tool rotation angle when the tool tip point is located at point *O*.

Finally, the trajectory of any cutting edge point *P(i*, *k)* located at a height $z_{i}$ on the edge *k*, as shown in Figure 8, is expressed in the system *O_W_X_W_Y_W_Z_W_* of the gear tooth (Figure 9) as a function of the previous transformation matrices **T***_W_*_1_(*f_x_*, *f_y_*, *f_z_*, *α*), **T**_12_(*γ*), **T**_23_(*β*) and **T**_3*T*_(*α*) and the coordinates of the cutting edge point *P(i*, *k)* in the tool system *O_T_X_T_Y_T_Z_T_* (Equations (14)–(16) in Step 5):

(33) $$\left[\begin{array}{c} x_{P\left(i,k\right)}^{W}\\ y_{P\left(i,k\right)}^{W}\\ z_{P\left(i,k\right)}^{W}\\ 1 \end{array}\right]{\;=\;T}_{W1}\left(f_{x}{,\;f}_{y}{,\;f}_{z},\;\alpha \right){\cdot T}_{12}\left(\gamma \right){\cdot T}_{23}\left(\beta \right){\cdot T}_{3T}\left(\alpha \right)\cdot \left[\begin{array}{c} x_{P\left(i,k\right)}^{T}\\ y_{P\left(i,k\right)}^{T}\\ z_{P\left(i,k\right)}^{T}\\ 1 \end{array}\right]$$

Once the cutting edge trajectories are obtained, surface topographies generated in each simulation area are obtained through the procedure described in Step 7.

**Figure 11:**
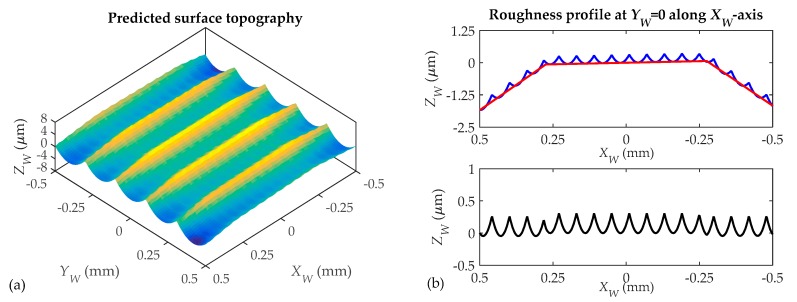

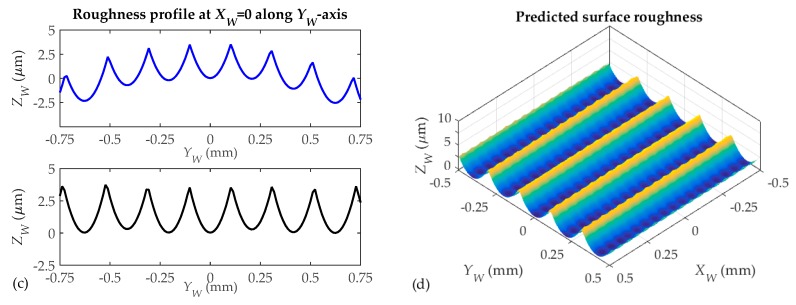
3D simulated surface topography (**a**); roughness profiles (**b**,**c**) and surface roughness (**d**): ball end mill with 3 mm diameter and *N_t_* = 3 cutting edges and feed value *f* = 0.18 mm/rev.

7. **Surface topographies determination (Figure 11).** The surface topography in the gear teeth is obtained from successive point positions of cutting edge points located on the *N_t_* edges with the tool rotating movement. The surface topography is simulated in a rectangular area defined along axes *X_W_* and *Y_W_*, as shown in Figure 7e. This area is divided into a discrete number of planes perpendicular to axes *X_W_* and *Y_W_*. For each plane, taking into account the trajectories of cutting edge points given by Equation (33), the model predicts the area swept by the tool cutting edges during the tool rotation and feed motion. The profile generated at each plane is obtained from the lowest positions of marks left by cutting edge points. By considering the profiles generated in those planes, the 3D surface topography in the rectangular area can be predicted. This allows the profiles generated along axes *X_W_* and *Y_W_* to be analyzed. As an example, Figure 11 shows the surface topography predicted for the simulation area selected in Figure 6b. In Figure 11a, a 3D representation of the predicted surface topography is shown. When the 2D profiles generated at two planes (*Y_W_* = 0 and *X_W_* = 0) are considered (Figure 11b,c), it can be observed that profiles are not only composed of roughness marks left by tool cutting edges. In Figure 11b, the straight sections defining the tool trajectory (red line) can be observed. In Figure 11c, the form of the gear tooth surface can also be observed. In order to analyze the surface roughness, the effect of tool trajectory in each milling pass is removed for each profile predicted along *Y_W_*-axis. As a consequence, roughness profiles (in black) are obtained. In Figure 11c, the form of the gear surface is also removed from the predicted profile and the roughness profile in black is obtained. In Figure 11d, the predicted surface roughness without the influence of the milling trajectory is shown. It can be observed that the step over between milling passes has a significant influence on the topography and the roughness peak-to-valley values. However, for this case, the effect of tool feed on roughness is less.

## 4. Model Validation Results

In order to validate the surface roughness predictive model explained in the previous section, measured roughness after gear machining, and model predicted roughness, were compared.

### 4.1. Gear Roughness Measurement

For measuring gear surface roughness (*R_a_* and *R_z_*) (Table 4), confocal tridimensional Leica^®^ DMC 3D and contact profilometer Taylor Hobson^®^ Form Taylorsurf were used for the 3D and 2D roughness measurement, respectively.

As it is mentioned in Section 3, the developed model predicts surface roughness in 5 different gear tooth zones. Nevertheless, surface roughness is only measured in two zones, always avoiding tooth edges (zones 1 and 5) and coinciding with the intermediate tooth zone. This fact must be taken into consideration when comparing predicted and measured roughness values. Therefore, for model validation, measured roughness values were compared to predicted values from zones 2–4.

### 4.2. Gear Roughness Prediction

The developed roughness model analyses 5 gear tooth zones and predicts surface roughness and generates surface topography and roughness profiles in each of these 5 zones. Thanks to this, different roughness profiles are predicted along tool feed direction *X_W_*. They are simulated for different tool edges number (*Nt*) and feed values (*f*) as can be seen in Figure 10 and Figure 11.

In Table 5, roughness values for each gear tooth flank depending on machining type, cutting patterns and programmed scallop height are shown. These values also depend on tooth flank (concave or convex), and on the analyzed zone (1–5). In Table 5, the first of the rows corresponds to the concave flanks and the second to convex ones.

In this case, taking into account that the selected gear design method corresponds to the Gleason method, the roughness values (*R_a_* and *R_z_*) are slightly different in the 5 analyzed zones. This is a consequence of the Gleason method, which generates a gear with a variable gearing height. For this reason, in the areas closest to the outer diameter, the obtained roughness values are higher.

The shape of the roughness profiles is a consequence of the straight sections between two points of the machining program (interpolation points) that define tool trajectory. The distance between the different interpolation points within the same path is approximately 0.56 mm.

**Figure 12:**
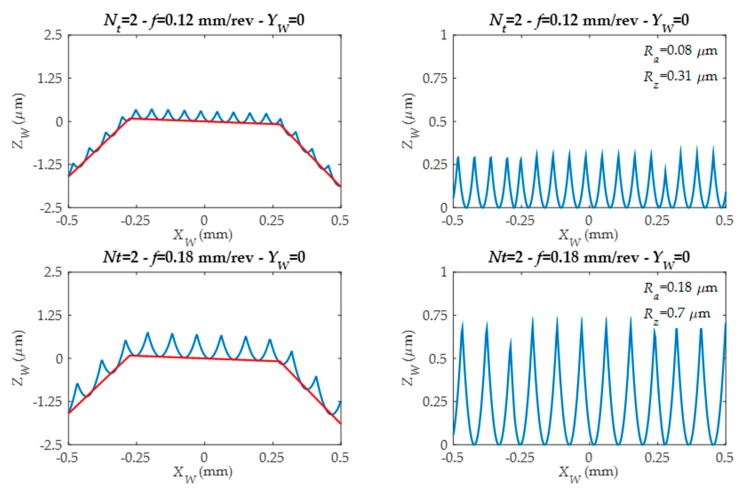
Predicted roughness profiles for different feed values and for the same tool.

**Figure 13:**
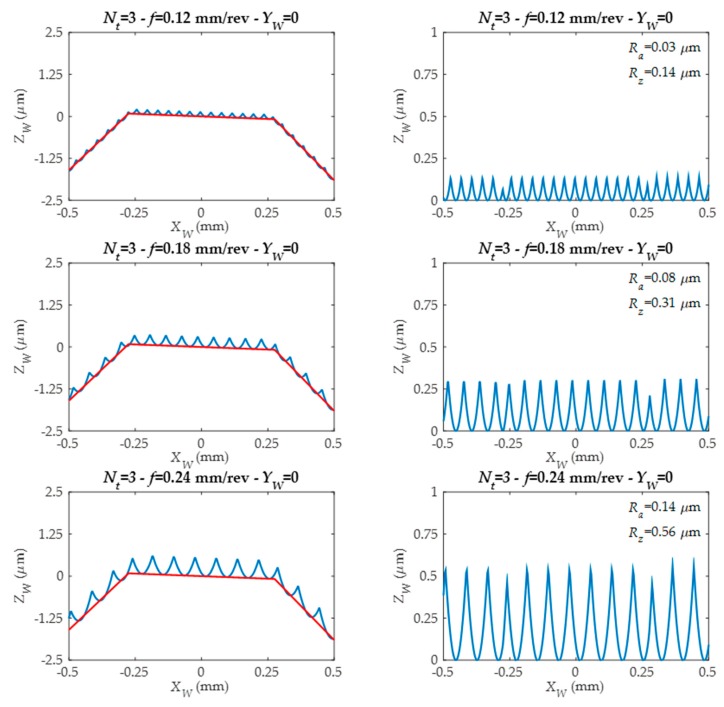
Predicted roughness profiles for tools with different edge numbers and working with different feed values.

In the left-side figures (Figure 12 and Figure 13), the straight sections defined by machining G1 (linear movement) are represented by a red line. The blue color represents the roughness profile. As can be seen, the roughness profile depends on the tool edge number (*N_t_*) and programmed tool feed value (*f*). In the right-side figures (Figure 12 and Figure 13), the form associated to workpiece component is eliminated, and for each roughness profile, both the arithmetic mean roughness parameter (*R_a_*) and the average roughness parameter (*R_z_*) are obtained.

### 4.3. Gear Roughness Predictive Model Validation

**Figure 14:**
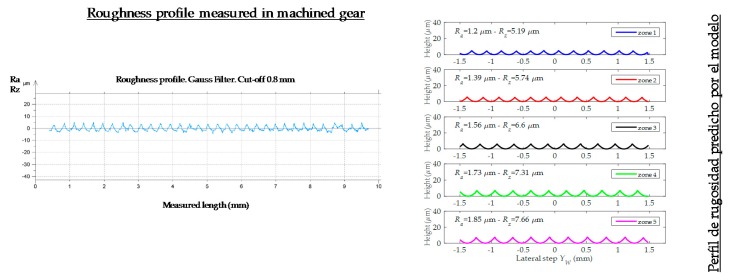
Roughness comparison of predictive model vs. measured roughness.

Figure 14 shows a comparison, for the same gear tooth, between the profile generated after gear measurement (after the machining process) and the profile generated by the developed roughness predictive model. The variations shown in some roughness profiles peaks correspond to the trajectory when interpolating between the different interpolation points that make up the same machining path.

## 5. Discussion

The obtained roughness results, which determine gear quality, agree with cited works related to gear manufacturing in CNC machines, and, indicate improved surface quality and process versatility. Moreover, more detailed results are added in this work for optimal machining strategies and predicting roughness.

There are several aspects to mention in relation to machining strategies, cutting parameters and roughness results. On one hand, for 3 + 2 axis machining, for both zig and zig-zag strategies, the obtained *R_a_* and *R_z_* values are slightly different for the zig strategy. The tool works down milling and machining time is increased due to non-cutting movements. Machining time is almost doubled. For 5 continuous axis machining, obtained results show differences that need to be taken into account. For zig cutting patterns, obtained roughness values for *R_a_* and *R_z_* are considerably lower than the ones obtained for zig-zag cutting patterns, about 50% lower. If both, 3 + 2 axis and 5 continuous axis machining are compared, better surface roughness values were obtained for 5 continuous axis machining with zig cutting pattern. On the contrary, the worst surface roughness results are obtained for 5 continuous axis machining with zig-zag cutting pattern. In relation to programmed stepovers, when programmed stepovers are reduced and the tool works up milling, the tool tends to go back to already machined path increasing roughness values. This is called ‘rail effect’ and is a consequence of tool deflection, which could explain why surface roughness values are higher for those machining trajectories with zig-zag cutting patterns.

The surface roughness prediction model was also used to analyze 5 axes and 3 + 2 axes machining strategies behavior. In the 3 + 2 axes machining case, the roughness values obtained from concave and convex gear flanks were similar. Instead, in the 5 continuous axis machining case, obtained surface roughness values were higher in convex gear flanks. Thanks to the developed roughness model, this problem can be corrected by changing the tool axis attack angle in the machining surface in order to improve surface roughness. Indirectly, the model also detects the so-called ‘rail effect’ previously mentioned, that is, the obtained roughness values almost doubled. The presented model also determined the influence of cutting parameters such as tool feed values (mm/rev) and tool edge number. Thus, it can be concluded, that for a higher tool edge number (maintaining tool feed values), the obtained roughness results were significantly lower. Clearly there is a direct link between programmed feed values and obtained roughness results. The results showed that a small increase in the feed value has a significant effect on the final surface quality obtained. After roughness values analysis in the tool feed direction and comparing these with those generated in the direction perpendicular to it, it is clear that the roughness due to the application of the different stepovers, for the same feed value programmed, is always higher. This makes it a more restrictive parameter, which is why it is measured in each of the different machined flanks.

## 6. Conclusions

In recent years, technological advances have enabled gear manufacturing in general purpose machines. This is a feasible process that is less limited to gear size and geometry than traditional technologies. The machining of gears in multitasking machines is presented here as a real application for this type of technology due to its flexibility, size and the variety of geometries that can be machined in this type of machine. It can be concluded that the presented roughness predictive model fulfills its function, making it possible to predict and control the cutting strategies and parameters to obtain the required surface finish. The use of standard tools for the machining of gears requires less time for tool supply, provides a greater variety range and competitive time and costs. In addition, it is possible to exchange tools between different machines. Specific gear manufacturing machines require ‘blank’ material for gear manufacturing. On the contrary, general purpose machines with multitasking technology enable the entire gear machining in a single machine and take a single reference in the workpiece. This ensures tight execution times and the required quality consecution. Therefore, the presented study has demonstrated that although spiral bevel gears geometry is a complex geometry, it is possible to machine it with 3 + 2 machining axes kinematics. The rotary axis can be positioned and fixed from the beginning for a more robust process. The obtained surface roughness values are acceptable in this case. Moreover, 3 + 2 machining is cheaper because less interpolated axes are required. Also, 3 + 2 axes programming is not as challenging as 5 axes programming. This work validates the developed surface topography model for a ball mill. There is concordance between experimentally obtained values and theoretical values obtained by the model. Moreover, the obtained values for different programmed stepovers maintain the same tendency. Topographical simulation becomes an essential tool after the programming of each of the finishing strategies because it optimizes machining results without resorting to the trial and error method. Thus, costs and time is reduced, which is desirable under current market conditions.

## Figures and Tables

**Table 1:** Spiral bevel gear geometry and parameters.

Spiral Bevel Gearing Design and Parameters		
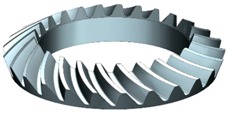	Gear heel pitch diameter (*Dp*)	200 mm
	Gear outside diameter (*De*)	207.6 mm
	Teeth number (*Z*)	25
	Spiral angle (*β*)	35°
	Face angle	59.5°
	Pressure angle (α)	20°

**Table 2:** Roughing strategy.

Roughing Strategy		Teeth Number	Tool
R-I	Cavity mill (3 + 1-axis) Follow periphery	1–25	Frontal mill Ø4 mm
R-II	Variable contour (5-axis) Stream line	1–25	Conical mill Ø3 mm

**Table 3:** Finishing strategies.

Finishing Strategy		Teeth Number	Tool
F-I	Surface area + Relative vector (3 + 2 axes) Cut pattern: ZIG (1–6) ZIG/ZAG(7–12) (Scallop 0.01–0.02–0.03 mm)	1–12	Conical mill Ø3 mm 8° Taper Ball nose TiAlN Number of flutes3 Helix angle 45°
F-II	Surface area + Relative vector (5 axes) Cut pattern: ZIG (13–18) ZIG/ZAG(19–25) (Scallop 0.01–0.02–0.03 mm)	13–25	

**Table 4:** Roughness measured results for gear teeth after finishing strategies.

Teeth Number	Machining	Strategy	Scallops (mm)	Measured Roughness Values	
				*R_a_* (µm)	*R_z_* (µm)
1	3 + 2 AXES	ZIG	0.01	1.68	8.64
2	3 + 2 AXES	ZIG	0.01		
3	3 + 2 AXES	ZIG	0.02	2.72	13.03
4	3 + 2 AXES	ZIG	0.02		
5	3 + 2 AXES	ZIG	0.03	4.66	20.83
6	3 + 2 AXES	ZIG	0.03		
7	3 + 2 AXES	ZIG-ZAG	0.01	1.91	10.12
8	3 + 2 AXES	ZIG-ZAG	0.01		
9	3 + 2 AXES	ZIG-ZAG	0.02	2.71	15.43
10	3 + 2 AXES	ZIG-ZAG	0.02		
11	3 + 2 AXES	ZIG-ZAG	0.03	4.83	22.20
12	3 + 2 AXES	ZIG-ZAG	0.03		
13	5 AXES	ZIG	0.01	1.34	7.78
14	5 AXES	ZIG	0.01		
15	5 AXES	ZIG	0.02	2.62	13.44
16	5 AXES	ZIG	0.02		
17	5 AXES	ZIG	0.03	4.58	19.63
18	5 AXES	ZIG	0.03		
19	5 AXES	ZIG-ZAG	0.01	4.24	20.03
20	5 AXES	ZIG-ZAG	0.01		
21	5 AXES	ZIG-ZAG	0.02	6.86	31.62
22	5 AXES	ZIG-ZAG	0.02		
23	5 AXES	ZIG-ZAG	0.02		
24	5 AXES	ZIG-ZAG	0.03	7.68	35.63
25	5 AXES	ZIG-ZAG	0.03		

**Table 5:** Roughness predicted results for gear teeth.

Teeth N°	Machining	Strategy	Scallop (mm)	Zone 1		Zone 2		Zone 3		Zone 4		Zone 5	
				*R_a_* (µm)	*R_z_* (µm)	*R_a_* (µm)	*R_z_* (µm)	*R_a_* (µm)	*R_z_* (µm)	*R_a_* (µm)	*R_z_* (µm)	*R_a_* (µm)	*R_z_* (µm)
1, 2	3 + 2 AXES	ZIG	0.01	1.2	5.19	1.39	5.74	1.56	6.6	1.73	7.31	1.85	7.66
				2.1	9	1.89	8.68	1.65	7.92	1.41	6.92	1.18	6.72
3, 4	3 + 2 AXES	ZIG	0.02	2.44	10.43	2.8	11.75	3.27	13.5	3.49	15.38	3.89	16.42
				4.33	18.71	3.82	16.37	3.39	14.47	2.88	13.15	2.36	11.1
5, 6	3 + 2 AXES	ZIG	0.03	3.72	16.39	4.31	18.6	4.93	20.16	5.42	22.35	5.7	24.45
				5.96	26.13	5.57	23.29	4.71	20.94	3.97	17.4	3.28	14.62
7, 8	3 + 2 AXES	ZIG-ZAG	0.01	1.19	5.16	1.35	5.77	1.65	9.29	1.66	7.07	1.83	7.75
				2.2	9.58	1.93	8.63	8.63	7.89	1.46	7.3	1.19	6.2
9, 10	3 + 2 AXES	ZIG-ZAG	0.02	2.28	9.8	2.63	11.44	11.44	14.38	3.34	17.21	3.47	15.01
				4.32	18.36	3.86	16.5	16.5	14.43	2.9	12.7	2.36	10.53
11, 12	3 + 2 AXES	ZIG-ZAG	0.03	3.41	14.75	3.96	16.95	16.95	19.71	5.07	20.07	5.35	19.98
				6.17	27.68	5.58	24.67	24.67	21.78	4.15	18.08	3.32	14.68
13, 14	5 AXES	ZIG	0.01	1.11	4.63	1.3	5.35	1.48	5.97	1.62	6.67	1.82	7.34
				2.3	9.68	2.19	8.93	1.93	8.21	1.68	7.42	1.46	6.52
15, 16	5 AXES	ZIG	0.02	2.25	9.2	2.59	10.52	2.95	11.93	3.32	13.39	3.6	14.66
				4.92	19.95	4.51	18.28	4.06	16.59	3.54	15.13	3.15	13.45
17, 18	5 AXES	ZIG	0.03	3.43	14.42	4.05	16.66	4.6	18.75	5.3	21.1	5.49	22.9
				7.16	29.17	6.56	26.78	5.84	25.04	1.58	23.39	4.89	20.33
19, 20	5 AXES	ZIG-ZAG	0.01	1.06	4.61	1.24	5.5	1.41	6.23	1.58	6.59	1.72	7.12
				2.5	9.61	2.22	8.63	2.02	7.67	1.92	7.35	1.59	6.37
21, 22, 23	5 AXES	ZIG-ZAG	0.02	2.22	8.34	2.47	9.59	2.86	11.05	3.25	12.44	3.43	13.34
				5.16	20.07	4.61	17.6	4.25	16.78	3.77	15.46	3.36	13.91
24, 25	5 AXES	ZIG-ZAG	0.03	3.2	11.76	3.71	13.62	4.27	16.91	4.87	19.54	5.37	20.01
				7.35	30.22	6.72	27.63	5.91	25.06	5.5	22.63	4.76	19.97
